# Supplementary figures and images for: p53 Promotes Cytokine Expression in Melanoma to Regulate Drug Resistance and Migration
Source: Cells. 2022 Jan 25;11(3):405. doi: 10.3390/cells11030405 (PMC8833998; doi:10.3390/cells11030405)

A

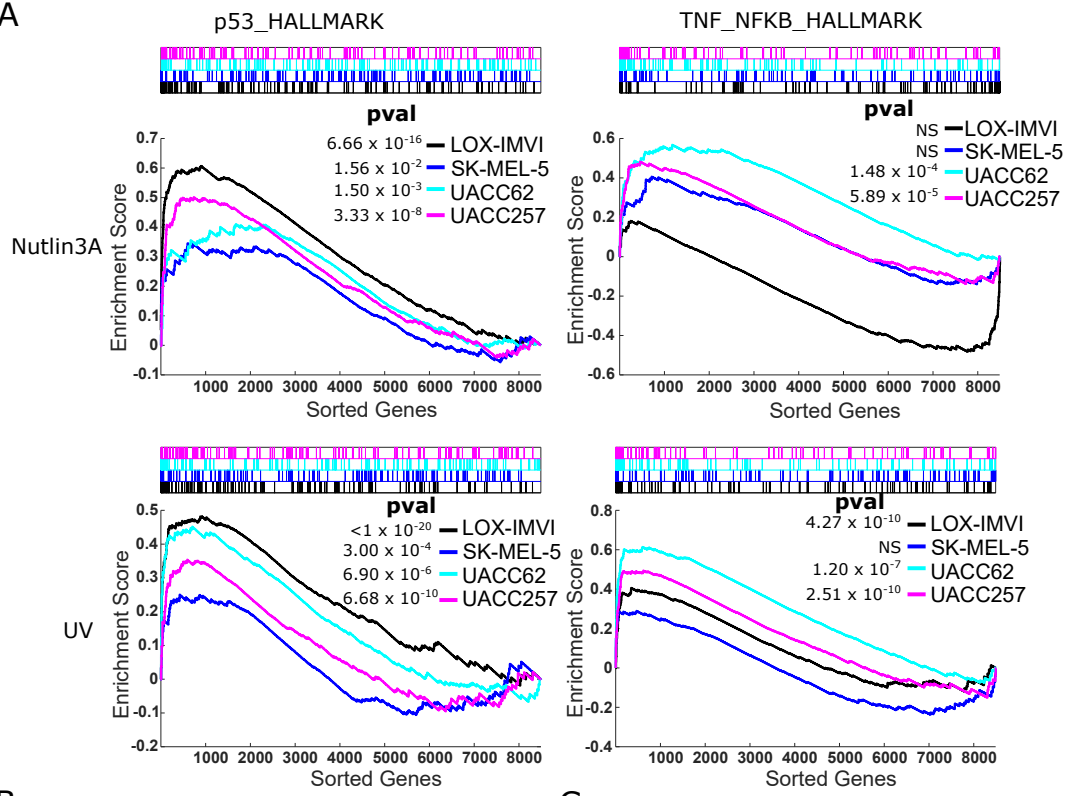

B

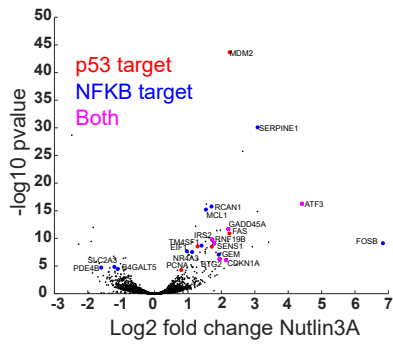

C

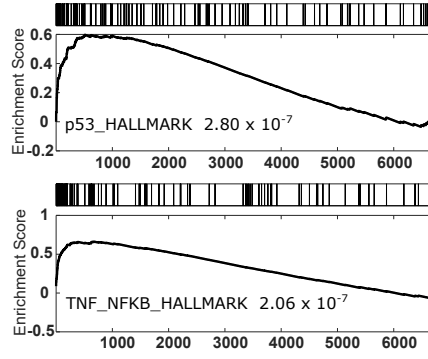

Supplement: Supplementary file 1 [file cells-11-00405-s001.zip › Figure S1_PR1.pdf]

A

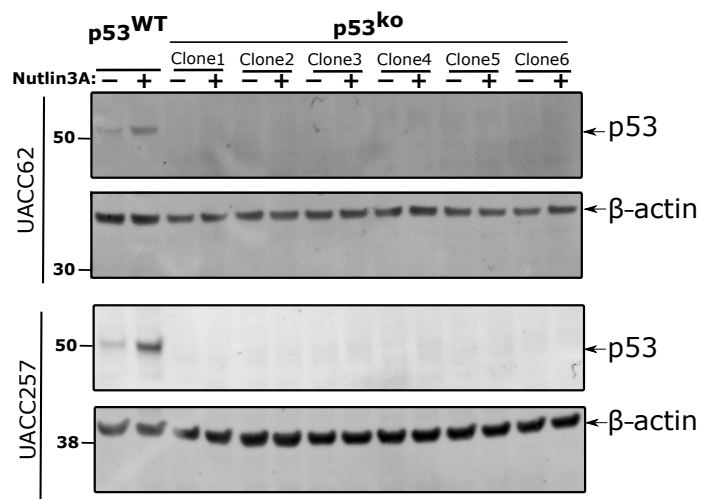

C

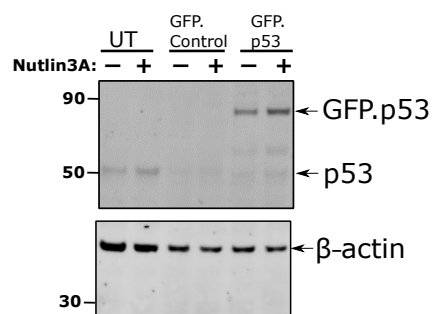

B

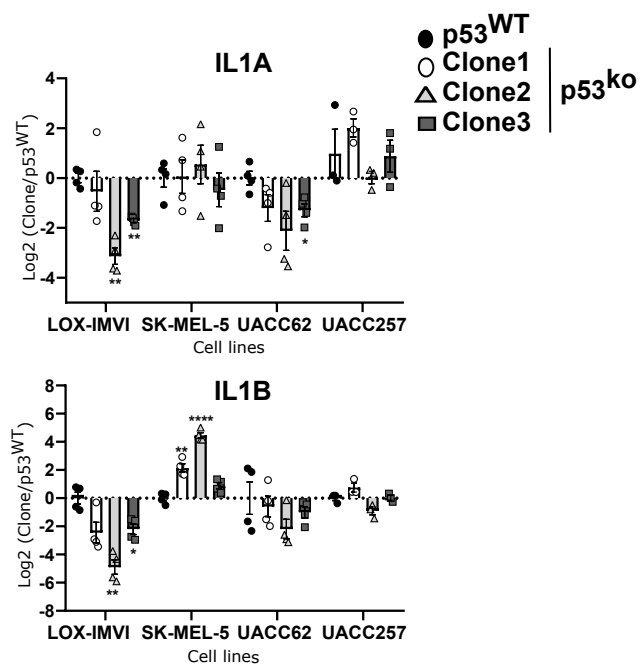

Supplement: Supplementary file 1 [file cells-11-00405-s001.zip › Figure S2_PR1.pdf]

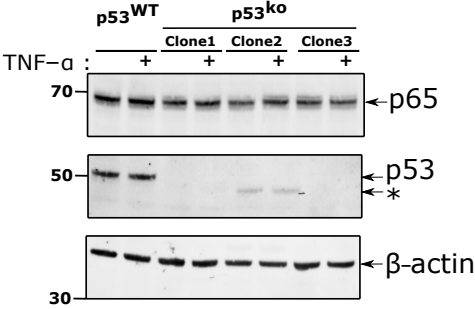

Supplement: Supplementary file 1 [file cells-11-00405-s001.zip › Figure S3_PR1.pdf]

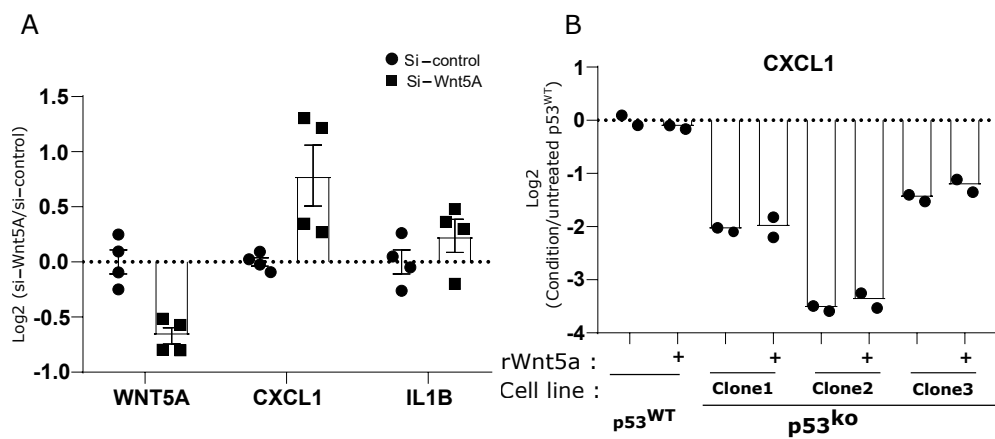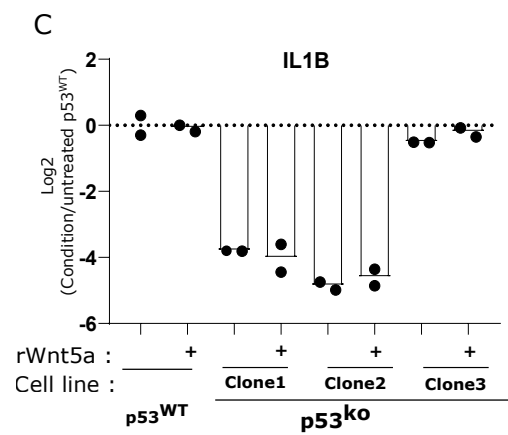

Supplement: Supplementary file 1 [file cells-11-00405-s001.zip › Figure S4_PR1.pdf]

A

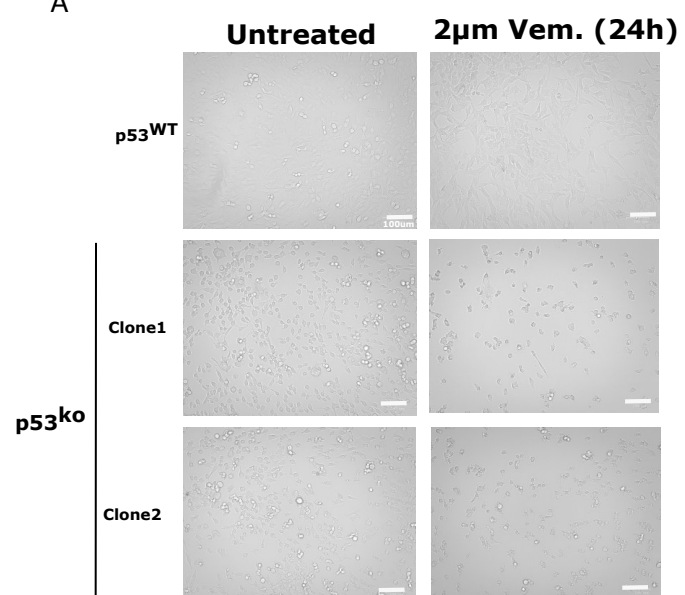

Supplement: Supplementary file 1 [file cells-11-00405-s001.zip › Figure S5_PR1.pdf]
